# Leucine-rich alpha-2-glycoprotein-1 is upregulated in sera and tumors of ovarian cancer patients

**DOI:** 10.1186/1757-2215-3-21

**Published:** 2010-09-10

**Authors:** John D Andersen, Kristin LM Boylan, Ronald Jemmerson, Melissa A Geller, Benjamin Misemer, Katherine M Harrington, Starchild Weivoda, Bruce A Witthuhn, Peter Argenta, Rachel Isaksson Vogel, Amy PN Skubitz

**Affiliations:** 1 Department of Laboratory Medicine and Pathology, University of Minnesota, MMC 609, 420 Delaware St. SE Minneapolis, MN, USA; 2 Department of Microbiology, University of Minnesota, Minneapolis, MN, USA; 3 Department of Obstetrics and Gynecology, University of Minnesota, Minneapolis, MN, USA; 4 Department of Biochemistry, Molecular Biology and Biophysics, University of Minnesota, Minneapolis, MN, USA; 5 Masonic Cancer Center Biostatistics and Informatics Core, University of Minnesota, Minneapolis, MN, USA

## Abstract

**Background:**

New biomarkers that replace or are used in conjunction with the current ovarian cancer diagnostic antigen, CA125, are needed for detection of ovarian cancer in the presurgical setting, as well as for detection of disease recurrence. We previously demonstrated the upregulation of leucine-rich alpha-2-glycoprotein-1 (LRG1) in the sera of ovarian cancer patients compared to healthy women using quantitative mass spectrometry.

**Methods:**

LRG1 was quantified by ELISA in serum from two relatively large cohorts of women with ovarian cancer and benign gynecological disease. The expression of LRG1 in ovarian cancer tissues and cell lines was examined by gene microarray, reverse-transcriptase polymerase chain reaction (RT-PCR), Western blot, immunocytochemistry and mass spectrometry.

**Results:**

Mean serum LRG1 was higher in 58 ovarian cancer patients than in 56 healthy women (89.33 ± 77.90 vs. 42.99 ± 9.88 ug/ml; p = 0.0008) and was highest among stage III/IV patients. In a separate set of 193 pre-surgical samples, LRG1 was higher in patients with serous or clear cell ovarian cancer (145.82 ± 65.99 ug/ml) compared to patients with benign gynecological diseases (82.53 ± 76.67 ug/ml, p < 0.0001). CA125 and LRG1 levels were moderately correlated (r = 0.47, p < 0.0001). *LRG1 *mRNA levels were higher in ovarian cancer tissues and cell lines compared to their normal counterparts when analyzed by gene microarray and RT-PCR. LRG1 protein was detected in ovarian cancer tissue samples and cell lines by immunocytochemistry and Western blotting. Multiple iosforms of LRG1 were observed by Western blot and were shown to represent different glycosylation states by digestion with glycosidase. LRG1 protein was also detected in the conditioned media of ovarian cancer cell culture by ELISA, Western blotting, and mass spectrometry.

**Conclusions:**

Serum LRG1 was significantly elevated in women with ovarian cancer compared to healthy women and women with benign gynecological disease, and was only moderately correlated with CA125. Ovarian cancer cells secrete LRG1 and may contribute directly to the elevated levels of LRG1 observed in the serum of ovarian cancer patients. Future studies will determine whether LRG1 may serve as a biomarker for presurgical diagnosis, disease recurrence, and/or as a target for therapy.

## Background

Ovarian cancer is the most lethal gynecologic malignancy [1]; about 22,000 women are diagnosed annually in the U.S. and ~16,000 patients succumb to the disease [2]. New biomarkers that either replace or are used in conjunction with the current ovarian cancer serum biomarker, CA125, are needed to improve diagnosis and treatment [1-4]. Biomarkers that distinguish between malignant and benign abdominal masses prior to surgery could identify those patients who should be referred to a gynecologic oncologist [5]. Initial cytoreductive surgery by a gynecologic oncology surgeon has been shown to result in improved outcomes for advanced ovarian cancer patients [6]. In addition, a biomarker that could be used to monitor the efficacy of therapy would be ideal to detect disease recurrence.

To date, serum biomarker discovery has been impeded by an abundance of twelve proteins that comprise ~95% of the serum proteome, and can mask lower abundance proteins [7]. We have previously reported the use of immunoaffinity depletion columns coupled with complementary mass spectrometry-based proteomic technologies to identify several differentially expressed proteins in the pooled sera of serous ovarian cancer patients compared to healthy women [8,9]. One such differentially expressed protein, leucine-rich α-2-glycoprotein-1 (LRG1), is ~3-fold more abundant in ovarian cancer serum compared to non-cancer control serum, and represents a potential serum biomarker for ovarian cancer.

Human LRG1 is a serum glycoprotein of 312 amino acids in length with a predicted unmodified molecular weight of 34 to 36 kD [10]. LRG1 has five potential glycosylation sites; 2 D SDS-PAGE results show LRG1 molecular weight ranges from 44 to 55 kD with isoelectric points ranging from 4.52 to 4.72 [11], suggesting that modifications occur. LRG1 has a normal plasma concentration of 21-50 μg/ml [12,13].

The function of LRG1 remains unknown, although reports have predicted its role in cell adhesion [14,15] due to its leucine-rich repeats, granulocytic differentiation due to its expression in neutrophil lineage experiments [16], and cell migration due to its overexpression in high-endothelial venules and tendency to bind extracellular matrix proteins [17]. LRG1 has been implicated as a protein involved upstream of the TGF-βR II pathway [18,19], suggesting a role in signalling. Serum LRG1 binding to cytochrome *c *has been recently demonstrated [20] and is proposed to play a role in cell survival and apoptosis [13,21].

In this study, we have validated our proteomic discovery experiments using sera, tissue, and cell lines from ovarian cancer patients and non-cancer controls.

## Methods

### Serum samples

Serum from patients with serous ovarian carcinoma (n = 58) and healthy female controls (n = 56) were obtained from the Gynecologic Oncology Group (GOG) Tissue Bank. The majority of ovarian cancer patients had stage III or IV serous tumors (n = 51), the others had stage I and II tumors (n = 7). The median age of the ovarian cancer patients was 52 years (range: 35-85 years) compared to 46 years (range: 19-58 years) for the non-cancer controls.

Additional sera were obtained from the University of Minnesota Tissue Procurement Facility (Minneapolis, MN). These samples were obtained immediately prior to surgery from women with suspected ovarian cancer. All patients were consented in accordance with the University of Minnesota Institutional Review Board (IRB) guidelines. Definitive diagnoses were determined by pathologists. A total of 193 samples were selected from patients with the following pathology: 10 benign mucinous ovarian cystadenomas, 10 fibromas, 19 cases of endometriosis, 16 cystadenomas, 30 other benign ovarian masses, 21 ovarian tumors of low malignant potential, 8 clear cell (5stage I or II; 3 stage III), and 79 serous ovarian cancers (11 stage I or II; 63 stage III or IV; 5 not staged). Collection, processing, and storage of all blood samples was strictly standardized as follows. Blood samples were collected in a vacutainer tube, allowed to clot at room temperature (RT) for 30 min, and centrifuged at ~2500 × g for 10 min at RT. The serum was removed and immediately divided into 100 μl and 1 ml aliquots, and stored at-80°C.

### Tissue and ascites samples

Tissue and ascites samples were obtained from the University of Minnesota Tissue Procurement Facility, as previously described [22,23]. All tissues were snap frozen in liquid nitrogen within 30 min of resection and stored in the vapor phase of liquid nitrogen. Tissue sections were made from each sample, stained with hematoxylin and eosin (H&E), and examined by a pathologist by light microscopy to confirm the pathological state of each sample; a second pathologist confirmed the diagnosis of each sample, documented the percent tumor (typically 100%), and documented any necrosis (typically none). The following tissues were analyzed in this study: 21 cases of serous ovarian cancer, 22 cases of serous ovarian cancer metastatic to the omentum, 24 cases of serous ovarian cancer widely metastatic to other regions (including peritoneal surfaces, bowel serosa, lymph nodes, liver, uterus, and the mesentery of the small bowel), 17 benign ovary tumors, 8 cases of ovarian tumors of low malignant potential, and 57 normal ovaries were analyzed for global gene expression. An additional 7 ovarian cancer and 13 normal ovary samples were used for RT-PCR and/or Western blot experiments. Ascites was obtained from 29 women undergoing surgery for the removal of serous ovarian cancer, as soon as it was released by pathology (typically within 1 hr of removal from the patient). Ascites was centrifuged at 600 × g for 10 min at RT, and the supernatant was immediately divided into small aliquots and frozen at -80°C.

### Cell Lines

Ovarian cancer cell lines SKOV3, ES-2, NIH:OVCAR3, HEY, C13, OV2008, OVCA429, OVCA433, A2780-S, and A2780-CP, provided by Dr. Barbara Vanderhyden (University of Ottawa, Canada); NIH:OVCAR5, provided by Dr. Judah Folkman (Harvard Medical School, Boston, MA); MA148 provided by Dr. Sundaram Ramakrishnan (University of Minnesota, Minneapolis, MN); CAOV3 provided by Dr. Robert Bast Jr. (University of Texas, Houston, TX) were maintained as previously described [24-26]. Immortalized normal ovarian surface epithelial (NOSE) cell lines 1816-575, 1816-686, HIO117, IMCC3, IMCC5, HIO3173-11, and HIO135, provided by Dr. Patricia Kruk (University of South Florida, Tampa, FL), were cultured as previously described [27,28]. All cells were maintained in a humidified chamber at 37°C with 5% CO_2 _and were routinely subcultured with trypsin/EDTA.

### Antibodies

Mouse IgG monoclonal antibody (mAb) 2F5.A2 against human sera LRG1 was used in the ELISAs [13]. Mouse IgG mAb 2E3 against recombinant human LRG1 (Abnova Corporation, Taipei, Taiwan) was used for Western blots and immunocytochemistry. Normal mouse IgG (Equitech-Bio, Inc. Kerville, TX) was used as a negative control for all experiments. Mouse mAb AC-74 against β-actin (Sigma Aldrich, St. Louis, MO) was used on Western blots as a loading control.

### ELISA

The ELISA for LRG1, which employs cytochrome c as the capture ligand, was conducted as described previously [13]. All samples were tested at least two times in triplicate. Concentrations of LRG1 were calculated from a purified standard [13]. The ELISA samples were compared as follows: for the GOG samples, mean LRG1 concentrations were compared across patients with ovarian cancer and control samples using general linear model for repeated measures, adjusted for age. For pre-surgical samples, mean LRG1 concentrations were compared across patients of the eight diagnoses using a general linear model for repeated measures and the least squared means are reported. T-tests were used to make comparisons between groups; all reported p-values are adjusted for multiple comparisons using a Bonferroni correction. CA125 levels were provided from the medical records. The CA125 levels were highly skewed and the log transformation was used for all analyses. Pearson's correlation was used to determine the association between CA125 and LRG1. The diagnostic value of LRG1, when used in addition to CA125, was considered using receiver operating characteristic (ROC) curves. ROC curves were constructed by plotting sensitivity versus 1-specificity and the areas under the curve (AUC) were calculated. Patients with a benign mass, mucinous ovarian tumors, fibroma, endometriosis and cystadenomas were defined as having benign pathology, patients with clear cell and serous ovarian cancer were defined as having cancer and patients diagnosed as having low malignant potential disease were excluded from the ROC analysis.

All values reported are means ± standard deviation (SD) unless otherwise noted. Statistical analyses were performed using SAS 9.2 (SAS Institute, Cary, NC).

### Gene Expression Analysis

Ovarian tissues from 149 patients were obtained from the Tissue Procurement Facility as described above; tissue samples were provided to Gene Logic Inc. (Gaithersburg, MD) for microarray analysis. On receipt of the tissue samples at Gene Logic Inc., H&E-stained slides were examined by a pathologist to verify the diagnosis and percentage of tumor tissue present, and the absence of necrosis. All tissue samples underwent stringent quality control measures to verify the integrity of the RNA before use in gene array experiments [22,23]. Total RNA was isolated and gene expression was assayed via the Affymetrix U133 Set gene array at Gene Logic Inc. Data was analyzed with the Gene Logic Genesis Enterprise System^® ^Software, using the Gene Logic normalization algorithm, as previously described [22,23]. The mean expression of LRG1 for each tissue type was calculated using the normalized expression values for Affymetrix probeset 228648_at, which is the only probe targeting LRG1 on this platform.

### Reverse Transcriptase PCR

Total RNA was isolated from cell lines and tissues as previously described [24]. The following oligos (Invitrogen, Carlsbad, CA) were used: *LRG1 *(forward, 5'CCATCTCCTGTCAACCACCT); reverse, 5'GTTTCGGGTTAGATCCAGCA) and *β-actin *(forward, 5'GGCCACGGCTGCTTC; reverse, 5'GTTGGCGTACAGGTCTTTGC). Select *LRG1 *cDNA amplicons were extracted, gel-purified, and sequenced with both *LRG1 *forward and reverse primers; sequences matched solely to *LRG1 *mRNA and genomic DNA sequences. As LRG1 is produced in the liver [29], we used liver mRNA as a positive control. β-actin served as a loading control.

### Protein Extraction

For tissue, ~ 100 mg of snap-frozen tissue was extracted using a PowerGen 125 hand-held homogenizer (Thermo-Fisher Scientific, Waltham, MA) in 2 ml of T-PER™ Tissue Protein Extraction Reagent (Thermo-Fisher Scientific) containing a serine-and cysteine-protease inhibitor cocktail (Roche Applied Science, Basel, Switzerland). Insoluble cellular components were removed by centrifugation at ~20,000 × g for 20 min. For cell lines, cells were grown to >90% confluency under normal conditions, rinsed twice with PBS and harvested with a rubber policeman. Cells were then pelleted at 7300 × g for 2 min and resuspended in 50 mM Tris-HCl, 150 mM NaCl, 0.1% (v/v) NP-40, pH 8.0; with Halt™ protease inhibitor cocktail, EDTA-free (Pierce Biotechnology, Rockford, IL). After a 30 min incubation on ice with intermittent vortexing, cell suspensions were sonicated at 20% duty cycle, output 2 with a Sonifier 450 analog (Branson Ultrasonics, Danbury, CT). Cellular debris was removed by centrifugation at 16000 × g for 20 min. Protein concentration was determined by the BCA method (Thermo-Fisher Scientific).

### Glycosidase Treatment

For deglycosidation, cell extracts or LRG1 purified from human plasma [13] were denatured and treated with Peptide: N-Glycosidase F (PNGase F) for 2 hr following the manufacturer's instructions (New England BioLabs, Ipswich, MA).

### Western Blotting

Protein samples in Laemmli buffer (2% SDS (w/w), 50% glycerol, 0.1 M DTT, 50 mM Tris, pH 6.8), were separated on a 4-20% or 10% Tris-HCl Criterion gel (Bio-Rad Laboratories, Hercules, CA), and electroblotted to a polyvinylidene difluoride (PVDF) membrane in 20% methanol, 25 mM Tris base, 192 mM glycine, pH 8.0. The PVDF membranes were blocked with 5% BSA in 20 mM Tris base, pH 7.6, containing 200 mM NaCl, and 0.05% Tween-20, and then incubated with primary antibodies at 1 μg/ml for 1 hr at RT. Membranes were then washed and incubated with a horseradish peroxidase-conjugated secondary antibody (Thermo-Fisher Scientific) and proteins were detected by enhanced chemiluminescence, using SuperSignal West Femto Maximum Sensitivity substrates (Thermo-Fisher Scientific) and exposed to film (Midwest Scientific, Valley Park, MO).

### Immunocytochemistry

Nineteen of 21 cell lines were examined by immunocytochemistry; the ovarian cancer cell line HEY and NOSE cell line IMCC5 were not analyzed. Cell lines were seeded into Nunclon™ 24 well plates (Nalge Nunc International, Rochester, NY) and grown to confluence. Cells were rinsed twice with PBS and then fixed with 100% methanol overnight at -20°C. Cells were rehydrated with PBS at RT and blocked with 5% v/v goat serum in PBS containing 0.1% Tween-20. Mouse mAb 2E3 (Abnova) against rLRG1 was added at a 1:50 dilution in blocking buffer and incubated overnight at 4°C. Cells were washed and incubated in a 1:50 dilution of fluorescein-labeled secondary antibody (goat polyclonal antibody against mouse heavy and light chains (IgG and IgM), Roche International, Basel, Switzerland) in the dark. Cells were washed, followed by incubation with 4', 6-diamidino-2-phenylindole (DAPI; Roche International) in blocking buffer. Cells were then washed with blocking buffer and stabilized with a SlowFade^® ^Antifade kit (Invitrogen, Carlsbad, CA). Cells were observed with an Olympus IX70 fluorescence microscope with a 20 × objective lens (Olympus, Tokyo, Japan) and a PixCell IIe™ Image Archiving Workstation camera (Molecular Devices, Sunnyvale, CA). Images were digitized using DVC View, v.2.2.8 software (DVC Company, Austin, TX). DAPI fluorescence was observed with a 285-330 nm excitation filter and a 420 nm absorption filter (U-MWU; Olympus). FITC fluorescence was observed with a 470 to 490 nm excitation filter and a 520 nm absorption filter (U-MP; Olympus).

### Processing of Serum-Free Conditioned Media

The ovarian cancer cell line NIH:OVCAR5, and the NOSE cell line, 1816-575, were grown to >90% confluency in media with serum [RPMI 1640 supplemented with L-glutamine, 0.2 U/ml bovine pancreas insulin (Sigma Aldrich), 50 U/ml penicillin and 50 μg/ml streptomycin (Mediatech, Inc., Manassas, VA) and 10% heat inactivated fetal bovine serum (FBS, Atlanta Biologicals, Lawrenceville, GA); or a 1:1 mixture of M199: MCDB 105 (Sigma Aldrich) supplemented with 0.1 mg/ml gentamicin (Invitrogen) and 15% FBS], as previously described [24-26]. Media was decanted and cells were rinsed three times with PBS. Cells were cultured for an additional 24 hr in serum-free MCDB 105 media (Sigma Aldrich). The media was collected and cellular debris was pelleted at 50,000 × g at 4°C for 1.5 hr. The media was concentrated using a 4 ml, 5000 MWCO PES membrane concentrator (VivaScience, Hanover, Germany) centrifuged at 5000 × g to a final volume of ~100 μl. Buffer exchange into PBS was accomplished by three reservoir changes with PBS. Protein concentration was determined by the BCA method.

### Mass Spectrometry

Proteins were subjected to tryptic digestion, dried down in a SpeedVac and rehydrated in water/ACN/FA (95:5:0.1). Mass spectrometry was performed on a linear ion trap (LTQ, Thermo Electron Corp., San Jose, CA). Peptide mixtures were desalted and concentrated on a Paradigm Platinum Peptide Nanotrap (Michrom Bioresources, Inc., Auburn, CA) precolumn (0.15 × 50 mm, 400-μl volume) and subsequently to a microcapillary column, packed with Magic C18AQ reversed-phase material on a flow splitter (Michrom Bioresources, Inc.) at ~250 nl/min. The samples were subjected to a 60 min (10-40% ACN) gradient and eluted into the microcapillary column set to 2.0 kV. The LTQ was operated in the positive-ion mode using data-dependent acquisition with (collision energy of 29%) on the top four ions detected in the survey scan. An inclusion list representing LRG1 (NCBI: gi|4712536) with *m/z *of +2 and +3 were included in the method.

### Database Searching

MS/MS samples were analyzed using SEQUEST (ThermoFinnigan, San Jose, CA) and X! Tandem http://www.thegpm.org. The search was done using an NCBI reference sequence of the Homo sapiens database (Oct, 2007; 33029 entries including known contaminants). The search parameters were carbamidomethyl-cysteine and oxidized methionine with 2 trypsin miscleavages. Scaffold (version Scaffold-01_05_14, Proteome Software Inc., Portland, OR) was used to validate MS/MS based peptide and protein identification. Protein probabilities were assigned by the Protein Prophet algorithm [30]. Proteins of interest with fewer than three peptides for ID were verified using manual inspection of product ion spectra in relation to candidate peptide sequences. Peptide candidates were judged as correct if a continuous series of a minimum of four b-or y-type product ions were present, if all product ion peaks were at least 3 times the intensity of background and if all experimental fragment ions could be matched to theoretical fragment ions.

## Results

### Quantification of LRG1 in Serum

**Figure 1:**
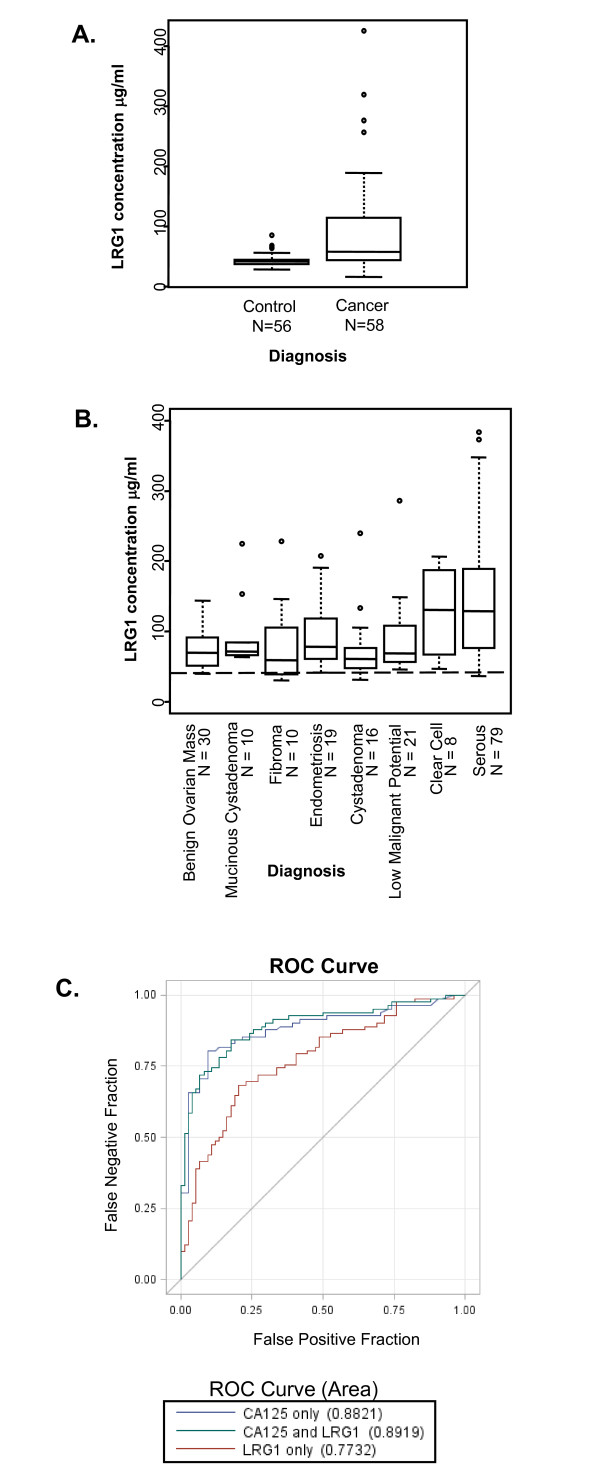
**ELISA detection of serum LRG1**. A) Serum LRG1 concentrations were determined for 58 ovarian cancer patients and 56 of the control patients. Box plots are presented here; the solid line indicates the median serum LRG1 for each group. Serum levels of LRG1 were significantly higher in the ovarian cancer sera than in control sera, after adjusting for age (p=0.0008). B) LRG1 in serum of individual patients with benign and malignant gynecological diseases. Median LRG1 values for each group are indicated by the solid bars. Dashed line indicates the mean LRG1 concentration from control serum in panel A. LRG1 concentrations are significantly higher in serum of women with ovarian cancer (serous and clear cell subtypes) than in serum of women with other gynecological diseases (p <0.0001). C) Receiver operator curves (ROC) for CA125 alone (blue line), LRG1 alone (red line) and LRG1 in combination with CA125 (green line). The area under the curve (AUC) for CA125 alone was 0.88, for LRG1 alone the AUC = 0.77, and the AUC for CA125 and LRG1 together was 0.89. There was no significant difference in sensitivity between CA125 alone and CA125 in combination with LRG1 (p=0.2728).

The level of serum LRG1 from 58 women with serous ovarian cancer and 56 healthy control women was quantified by ELISA. The distribution of serum LRG1 levels and age of patients and controls is presented in Table 1. Ovarian cancer patients had a statistically significant ~2-fold increase in serum LRG1 compared to healthy controls (age adjusted, p = 0.0008; Figure 1A). The mean LRG1 concentration for ovarian cancer patient sera was 89.33 ± 77.97 μg/ml compared to 42.99 ± 9.88 μg/ml for non-cancer sera. Because the age of the ovarian cancer group was significantly higher than that of the healthy controls, we further explored the effect of the age difference between cases and controls and found age did not affect the significant difference in LRG1 concentration between the cancer and control groups (results not shown). When the 58 ovarian cancer serum samples were separated by stage, the mean LRG1 serum level for the stage I and II cancer patients (n = 7) was 62.52 ± 36.53 μg/ml, compared to 93.01 ± 81.50 μg/ml for the stage III and IV cancer patients (n = 51, p > 0.05).

**Table 1:** LRG1 concentration in sera from serous ovarian cancer patients and healthy female controls

	N	Median Age	LRG1 μg/ml
**Total**	114		
Control	56	42.00	42.99 +/- 9.88
Cancer	58	64.00	89.33 +/- 77.90

**Cancer Stage**			
1, 2	7	57.00	62.52 +/- 36.53
3, 4	51	65.00	93.01 +/- 81.50

ELISAs were then performed on a second set of individual serum samples from women taken pre-surgery for a gynecologic disease (Table 2). Among the eight diagnosis groups, the 79 serum samples from women with serous ovarian cancer had the highest mean LRG1 level (135.54 ± 64.16 μg/ml), closely followed by the 8 serum samples from women with clear cell cancer (134.26 ± 61.18 μg/ml). LRG1 concentrations were significantly different across diagnosis groups (p < 0.0001, Figure 1B). After adjusting for multiple comparisons, the most notable difference was between serous ovarian cancer and other benign ovarian mass (p = 0.0007), with LRG1 concentrations being significantly higher in the serous ovarian cancer patients. All of these LRG1 levels were higher than those of the non-cancer healthy controls from the original set of sera tested (Figure 1A).

**Table 2:** Concentration of LRG1 in sera collected prior to surgery

Diagnosis		N		Mean^1 ^ [LRG1] μg/ml		95% CI		N		Mean Age		95% CI		N		Log (CA125)		95% CI
Serous		79		135.54		121.30, 149.78		79		64.03		61.36, 66.69		74		6.21		5.87, 6.55
Clear Cell		8		134.26		91.59, 176.93		8		58.38		50.00, 66.75		8		4.53		3.49, 5.57
LMP		21		91.11		64.17, 118.05		20		51.40		46.10, 56.70		16		4.62		3.88, 5.35
Mucinous Cystadenoma		10		94.31		55.63, 132.98		10		45.60		38.11, 53.09		8		3.35		2.31, 4.39
Benign Ovarian Mass		30		71.76		47.18, 96.34		27		52.15		47.59, 56.71		25		2.91		2.32, 3.50
Cystadenoma		16		73.06		42.47, 103.65		16		53.00		47.08, 58.92		14		3.21		2.43, 4.00
Endometriosis		19		87.49		59.06, 115.93		19		43.11		37.67, 48.54		18		4.25		3.56, 4.94
Fibroma		10		88.23		50.28, 126.17		10		63.20		55.71, 70.69		9		3.50		2.52, 4.48

**Total**		**193**						**189**						**172**				

^1 ^Least-squares means from repeated measures general linear model.

We found a moderate correlation between CA125 and LRG1 (r = 0.47, p < 0.0001). In order to examine the diagnostic value of LRG1 in distinguishing patients with benign tumors from those with ovarian cancer, we compared receiver operator curves (ROC) for CA125 alone, LRG1 alone and in combination with CA125 (Figure 1C). The ROC of the combined markers was not significantly different from the ROC of CA125 alone; the area under the curve (AUC) for CA125 alone was 0.88 (95% CI: 0.82, 0.94) and the AUC for CA125 and LRG1 was 0.89 (95% CI: 0.84, 0.96; p = 0.2728). There was no significant improvement in sensitivity when adding LRG1.

Ascites fluid from 29 women with serous ovarian cancer was also tested by ELISA for LRG1 protein and was found to be elevated relative to serum levels with a mean value of 142.28 ± 73.56 μg/ml.

### Differential Expression of *LRG1 *mRNA

**Figure 2:**
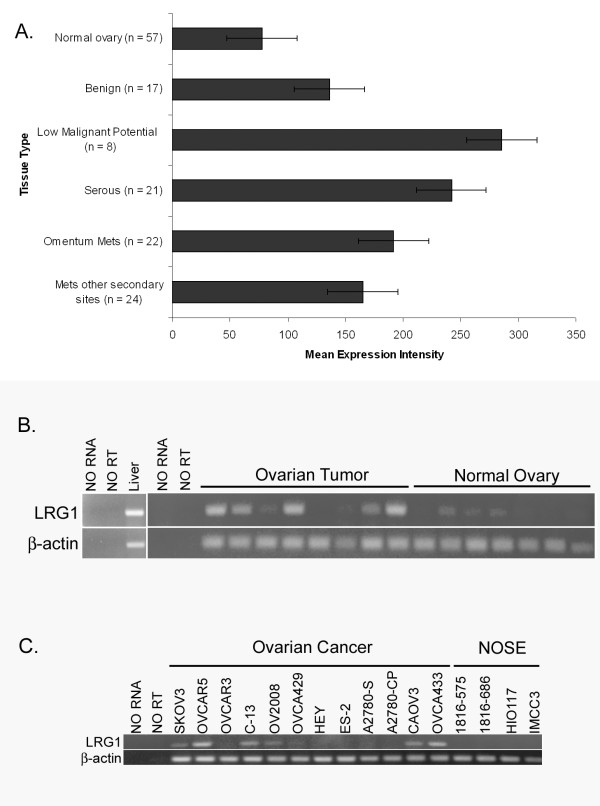
**Expression of *LRG1 *transcripts in ovarian cancer tissues and cell lines**. A) Microarray analysis of LRG1 gene expression in ovarian cancer tissues was performed on Affymetrix HU_133 gene chips. Mean expression of LRG1 RNA was determined for normal ovary, benign ovary tumors, and primary and metastatic ovarian cancers. (n) = number of samples per tissue type. B) RT-PCR of LRG1 expression in ovarian cancer tissue samples (N = 8) relative to normal ovary tissue (N =7). C) LRG1 expression in ovarian cancer cell lines compared to immortalized NOSE cell lines. β-actin was used as an amplification control.

To determine whether the ovarian cancer cells may serve as a potential source of the increased serum LRG1 levels in ovarian cancer patients, we quantified *LRG1 *mRNA expression in ovarian tumors compared to normal ovaries by gene microarray analysis (Figure 2A). *LRG1 *mRNA expression levels were about 2-fold higher in benign ovarian tumors and about 3-4 fold higher in ovarian serous cancers compared to normal ovaries. Similarly, *LRG1 *expression levels were ~2 to 2.5-fold higher in ovarian tumor metastases than in normal ovaries (Figure 2A). Interestingly, although a small sample size, the highest LRG1 mRNA levels were in tumors of low malignant potential.

Using RT-PCR, we also detected increased *LRG1 *mRNA expression in ovarian tumors compared to normal ovaries (Figure 2B). Eight tissue samples from patients with stage II or higher serous ovarian cancer and seven normal ovaries were tested. Six of the eight ovarian cancers expressed higher levels of *LRG1 *mRNA than normal ovaries. As LRG1 is an acute-phase protein, primarily produced in the liver [29], we used liver mRNA as a positive control.

To control for the possible influence of stromal, endothelial, and blood cells present in tissue samples, we examined *LRG1 *mRNA expression levels in ovarian cancer and NOSE cell lines by RT-PCR. *LRG1 *mRNA expression was observed in 7 of the 12 ovarian cancer cell lines tested, but no measurable expression was detected in the 4 immortalized NOSE cell lines (Figure 2C).

### Differential Expression of LRG1 Protein

**Figure 3:**
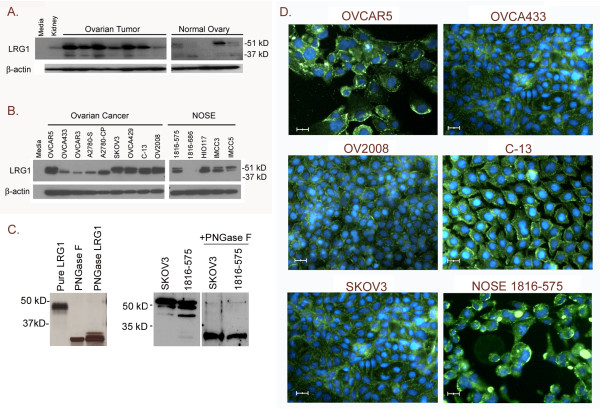
**Expression and localization of LRG1 protein in ovarian cancer tissues and cell lines.** A) 50 µg of total protein extract from ovarian cancer tissues (N = 7) and normal ovaries (N =5) were evaluated by Western blot for LRG1 protein expression. Kidney was used as a positive control tissue, as it contains an abundance of epithelial cells. B) LRG1 protein expression in 20 µg of total protein extract from ovarian cancer cell lines and immortalized NOSE cells. β-actin was used as the loading control. C) Left panel; silver stained polyacrylamide gel of LRG1 purified from human plasma, PNGase F, and purified LRG1 treated with PNGase F. Right panel; Western blot for LRG1 in protein extracts from cell lines with and without PNGase F treatment. D) Subcellular localization of LRG1 is shown by immunocytochemistry in ovarian cancer cell lines (OVCAR5, OVCAR433, OV2008, C-13, and SKOV3) and immortalized NOSE cell line (1816-575); 200X magnification, scale bar = 20 µm. FITC (green) = LRG1, DAPI (blue) = nucleus.

Western blotting was used to determine if LRG1 protein was present at higher levels in serous ovarian cancer tissues compared to normal ovaries. All seven ovarian cancer specimens demonstrated higher levels of LRG1 protein than the five normal ovaries (Figure 3A). Although several protein bands were visualized in both the ovarian cancer tissues and the normal ovary, the size of the major protein band in the tumors was ~47 kD, while the major protein band in normal ovaries was ~ 51 kD. A minor protein band of ~34-36 kD, which corresponds to the predicted size of unmodified LRG1, was observed in several of the tumor and normal ovary samples. A single protein band at ~47 kD was visualized in normal kidney tissue (Figure 3A) and also in liver tissue (not shown).

Because surface epithelial cells comprise only a minor fraction of the normal ovary, we also examined the expression of LRG1 protein in cell lines derived from ovarian cancer cells and normal ovarian surface epithelia. In Western blot analysis of cell lines, the ~47 and ~51 kD forms of LRG1 protein were present in both ovarian cancer and NOSE cell lines (Figure 3B); the predominant form detected in all cases was 47 kD. Interestingly, four of the five serous ovarian cancer cell lines, OVCA433, OVCAR3, A2780-S, and A2780-CP expressed predominantly the ~47 kD form of LRG1 and little to none of the ~51 kD protein band. Two other serous ovarian cancer cell lines, CAOV3 and MA148, also expressed high levels of the ~47 kD band, but not the ~51 kD band (data not shown). In addition, the cisplatin-resistant cancer line A2780-CP expressed higher levels of the ~47 kD protein band compared to its cisplatin-sensitive counterpart A2780-S (Figure 3B). No LRG1 protein was detected in the NOSE cell line 1816-686.

To establish whether the multiple iosforms of LRG1 observed by Western blot represent different glycosylation states, we treated purified LRG1 protein and cell-free extracts with the enzyme PNGase F to remove carbohydrate residues from the LRG1 protein backbone. As shown in Figure 3C (left panel), LRG1 purified from human plasma has an apparent molecular weight of ~ 47 kD prior to PNGase F treatment. After digestion, the molecular weight of LRG1 is reduced to ~ 34 kD, indicating protein deglycosylation. Similar results were observed in cell-free extracts of the ovarian cancer cell line SKOV3 and the NOSE cell line 1816-575 (Figure 3C, right panel), where multiple higher molecular weight species were reduced to a single lower molecular weight band upon digestion with PNGase F.

### Cellular Localization of LRG1

Using immunocytochemistry, LRG1 protein was detected in the cytoplasm of all 19 cell lines tested; representative examples are shown in Figure 3D. LRG1 also localized to the plasma membrane in most of the ovarian cancer cell lines. Three NOSE cell lines (HIO135, HIO117, and IMCC3) also had moderate amounts of LRG1 localized to the plasma membrane. Punctate cytoplasmic localization was observed in NIH:OVCAR5, HEY, C-13, OV2008, ES-2, and OVCA429 ovarian cancer cell lines and all six of the NOSE cell lines. Consistent with the Western blot, the cisplatin-resistant cancer line A2780-CP demonstrated more intense staining compared to its cisplatin-sensitive counterpart A2780-S (data not shown).

### Identification of LRG1 in NIH:OVCAR5 Conditioned media

**Figure 4:**
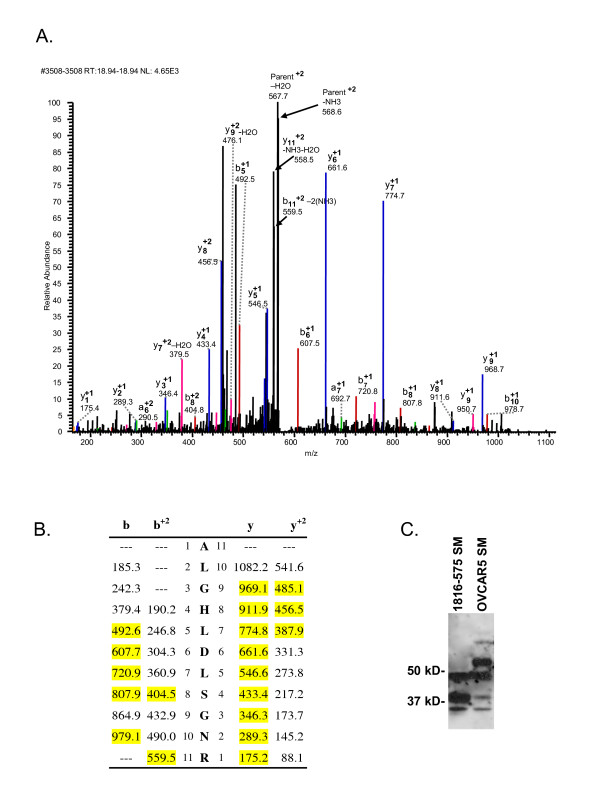
**Secretion of LRG1 into conditioned media by ovarian cancer cell line NIH:OVCAR5**. A) MS spectrum for LRG1 peptide, ALGHLDSGNR, identified in the spent media of NIH:OVCAR5 cells with 95% (peptide) probability. Conditioned media from the ovarian cancer cell line was concentrated and processed for MSMS analysis. LRG1was identified with low (protein) probability with a single peptide in the complex mixture. The identity of the peptide was confirmed by manual inspection. Peak assignments are indicated. B) m/z for predicted b- and y- ions for peptide ALGHLDSGNR. Highlighted peaks were identified in the spectrum shown in A. C) Western immunoblot of conditioned media from NOSE cell line 1816-575 and ovarian cancer cell line NIH:OVCAR5. 50 µg of concentrated, conditioned media from each cell line was loaded. Position of molecular weight standards, left.

To determine whether ovarian cells secrete LRG1 and thus may directly contribute to the elevated levels of LRG1 protein observed in the ovarian cancer patients' sera, we analyzed serum-free conditioned media from NIH:OVCAR5 cells using mass spectrometry. We have previously identified twelve LRG1 peptides in serum by the mass spectrometry-based proteomic techniques of iTRAQ^® ^and DIGE (Table 3; [8,9]). Three of these peptides, DLLLPQPDLR, ALGHLDLSGNR, and YLFLNGNK, were detected in sera in multiple experiments (Table 3; [8,9]). Similarly, using an inclusion list of all predicted tryptic LRG1 peptides, we used mass spectrometry to identify the LRG1 peptide ALGHLDLSGNR at 95% confidence (Scaffold score) in NIH:OVCAR5 conditioned media; the peptide identity was confirmed by manual inspection of the mass spectrum (Figure 4). The peptide ALGHLDLSGNR is unique to human LRG1, which supports the idea that LRG1 is produced and secreted by the NIH:OVCAR5 cells rather than being introduced from the growth media.

**Table 3:** LRG1 peptides identified by mass spectrometry

Depletion experiment	# of unique peptides	Peptide sequence	Peptide sequence confidence	Sequence coverage	*m/z*
MARS SC**†**	3	TLDLGENQLETLPPDLLR	99	192-209	2037.29
		DLLLPQPDLR	31	230-239	1179.37
		VTLSPK	N/A	36-41	643.76

IgY-12 SC**†**	6	LQELHLSSNGLESLSPEFLRPVPQ	99	94-117	2691.03
		ALGHLDLSGNR	99	165-175	1152.26
		TLDLGENQLETLPPDLLR	99	192-209	2037.29
		DLLLPQPDLR	98	230-239	1179.37
		LQVLGK	27	224-229	656.81
		YLFLNGNK	13	240-247	968.1

IgY-12 LC**†**	10	ALGHLDLSGNR	99	165-175	1152.26
		TLDLGENQLETLPPDLLR	99	192-209	2037.29
		VAAGAFQGLR	99	251-260	989.13
		GQTLLAVAK	99	337-345	900.07
		DLLLPQPDLR	98	230-239	1179.37
		LHLEGNKLQVLGK	97	217-229	1448.71
		YLFLNGNK	89	240-247	968.1
		GPLQLER	81	210 216	811.92
		LQVLGK	24	224-229	656.81
		VLDLTR	8	120-125	715.84

IgY-12 LC**‡**	6	VAAGAFQGLR	95	251-260	989.13
		YLFLNGNK	95	240-247	968.1
		ALGHLDLSGNR	95	165-175	1152.26
		GQTLLAVAK	95	337-345	900.07
		DLLLPQPDLR	95	230-239	1179.37

† iTRAQ^® ^labeling; ‡Differential in-gel electrophoresis labeling.

LRG1 was also detected in the conditioned media of the NIH:OVCAR5 cells by Western blotting. We observed two major LRG1 protein bands of ~47 and ~51 kD, as well as minor protein bands of ~34/36, ~39/40, and ~65 kD in the NIH:OVCAR5 conditioned media (Figure 4C). By comparison, Western blots of the conditioned media from the NOSE cell line 1816-575 detected major LRG1 protein bands of ~47 and ~39/40 kD as well as a minor protein band of <37 kD. Finally, we used the ELISA to detect LRG1 in the NIH:OVCA5 conditioned media (data not shown). Taken together, these results demonstrate that, in addition to being synthesized in the liver, ovarian cancer cells synthesize and secrete LRG1, and may therefore contribute to the elevated LRG1 levels observed in the sera of the ovarian cancer patients.

## Discussion

We recently identified leucine-rich alpha-2-glycoprotein-1 (LRG1) as one of several proteins overexpressed in the serum of patients with ovarian cancer [8,9]. In this study, we sought to validate this observation and quantitate the levels of LRG1 in a larger cohort of patients' sera. We have also shown that ovarian cancer cells may directly contribute to the elevated levels of LRG1 observed in patients' sera.

The increased serum LRG1 levels in ovarian cancer patients that we had observed by Western blot in pooled samples [8], were also evident by ELISA in individual samples. When the initial 114 serum samples were tested by ELISA, serum LRG1 was found to be approximately 2-fold greater in serous ovarian cancer patients' sera compared to sera from healthy control women; however, the variance among the ovarian cancer patient samples resulted in unfavorable estimates of sensitivity and specificity. This led us to explore the levels of serum LRG1 among women with different types of benign and malignant ovarian masses. Using a separate set of 193 patient serum samples obtained immediately prior to surgery for a suspicious adnexal mass, LRG1 values were significantly higher (1.7-fold) in patients with serous and clear cell ovarian cancer compared to those with benign gynecological diseases. Although our gene microarray data showed that LRG1 mRNA expression levels were greatest in low malignant potential tumors, the level of serum LRG1 protein in the LMP tumors was significantly lower than for both serous and clear cell ovarian cancer.

Although a biomarker for the early detection of ovarian cancer would have a greater impact, the ability to distinguish malignant from benign disease prior to surgery could be useful in determining which patients would benefit from treatment by a gynecological oncologist. Recently, a panel of biomarkers was approved by the FDA to aid in the diagnosis of ovarian tumors prior to surgery (OVA1; [5]). This panel includes CA125 as well as beta-2 microglobulin, apolipoprotein A1, transthyretin and transferrin, but not LRG1.

Though the mean concentration of serum LRG1 in serous ovarian cancer patients differed between samples in the two data sets, differences in serum preparation and storage may have affected the quantity of LRG1 detected. For example, Govorukhina et al., [31] recently reported that LRG1 levels were decreased in serum with clotting time of longer than 1 hr. We maintained a strict protocol for sample collection and storage for the samples taken from patients at the University of Minnesota, in order to minimize these types of variations (see Methods), and this likely explains the higher LRG1 values in the second dataset compared with the first set of samples obtained from the GOG.

Initially, LRG1 was classified as an "acute-phase protein" involved in the body's response to bacterial and viral infection [32], but has since been identified as elevated in a variety of disease states, both malignant and benign, including toxic-shock syndrome [13], and during inflammatory responses of cystic fibrosis [33]. LRG1 is also increased in serum of patients with hepatocellular carcinoma following therapeutic ablation treatment [34]. Differential expression techniques employing affinity depletion of high abundance proteins and 2 D electrophoresis have found serum LRG1 to be upregulated in lung and pancreatic cancer [35-37]. Proteomic research using 2 D SDS-PAGE to analyze body fluids found LRG1 to be upregulated in cerebrospinal fluid and serum of patients with hydrocephalus and silicosis [19,38].

We conducted a series of experiments examining ovarian cancer tumor cells as a possible source of serum LRG1. Others have identified LRG1 peptides by mass-spectrometry in the secreted or cell surface fractions of CAOV3 and OVCAR3 serous ovarian cancer cell lines, but not in the clear cell ovarian cancer cell line ES-2 [39]. LRG1 peptides have also been identified in ascites fluid and cells from ovarian cancer patients [40]. Recently, elevated levels of LRG1 have been identified in chemoresistant ovarian tumor tissue [41], and in immunodepleted serum, using ICAT quantitative proteomics [42]. Additionally, LRG1 peptides have been identified in the conditioned media of prostate cancer [43,44], and breast cancer cell lines [45] and in the peritoneal fluid of women with uterine leiomyomas [46]. The production and secretion of LRG1 by tumor cells suggests there may be a more direct relationship between tumor burden and serum levels of LRG1 than for other acute phase proteins secreted only by the liver. For example, although haptoglobin levels are increased in the sera of ovarian cancer patients, no haptoglobin RNA or protein were detected by Ye et al. [47] in seven ovarian cancer cell lines.

In a limited number of cases, we have analyzed sera from patients prior to surgery and following treatment for ovarian cancer. We have found that serum LRG1 levels appear to be more directly related to tumor burden compared to CA125. For example, in three of six patients with sub-optimal debulking surgery, CA125 levels dropped substantially, while LRG1 levels remained elevated. In six cases, serum LRG1 dropped dramatically post chemotherapy. In five cases, LRG1 levels appeared to rise prior to CA125 levels and the onset of recurrent disease. However, given the very low numbers of patients that we have analyzed to date, the use of LRG1 as a marker for disease recurrence, while tantalizing, is purely speculative.

By immunocytochemistry, LRG1 was localized to the cytoplasm of all of the ovarian cell lines tested, both cancer and normal, and was observed on the plasma membrane of most. The serous papillary ovarian cancer cell line, NIH:OVCAR5, had the most intense plasma membrane staining for LRG1. In addition, this cell line expressed high levels of the ~51 kD LRG1 protein band. The LRG1 sequence contains a predicted transmembrane domain [48] which overlaps the signal sequence; this may allow for the expression of LRG1 at the cell surface. Alternatively, the localization of LRG1 that we observed on the surface of the NIH:OVCAR5 cells may be indicative of cells in the process of secreting LRG1.

Examination of ovarian tumor extracts and cell lines by Western blot revealed increased expression of LRG1 protein in malignant serous tumors and ovarian cancer cell lines compared to their respective controls, as well as the presence of several isoforms of LRG1, though notably the ~47 kD LRG1 band was most intense in each of the malignant ovarian tumor protein extracts. The presence of numerous isoforms for LRG1 has previously been shown by 2 D SDS-PAGE [8,11,32,34,36-38], suggesting the presence of multiple glycosylated isoforms of LRG1. Indeed, we showed that glycosidase treatment of LRG1, both purified and in extracts of ovarian cancer and NOSE cell lines, reduced the apparent molecular weight of LRG1 indicating the presence of carbohydrate modifications of the protein backbone.

The N-glycosylation of LRG1 produced by the ovarian cancer cells is consistent with its secretion. The ~51 kD band was found at very low levels in the ovarian cancer tumor extracts and was present in the protein extracts of only a few of the ovarian cancer cell lines. It is possible that this ~51 kD glycoform of LRG1 is secreted by the serous ovarian cancer cells, and may contribute to the elevated levels of LRG1 quantitated by ELISA in the sera of these patients. This hypothesis is supported by our Western blot findings that an ~51 kD band was found in the conditioned media of the NIH:OVCAR5 cells but not the NOSE cells, again suggesting that the ~51 kD glycoform of LRG1 may be preferentially secreted, or aberrantly glycosylated in ovarian cancer.

Glycosylation of serum proteins in cancer states is well documented, and serum glycoproteins are being investigated for use as biomarkers in prostate, breast, lung, ovarian and other gynecologic cancers [49-52]. Glycosylation of surface proteins on ovarian carcinoma cells has been reported to mediate adhesion, migration, and invasion through the ECM [53]. Given that murine LRG1 has been shown to bind to several extracellular matrix proteins, and also TGFβ [17], a possible role for LRG1 in ovarian cancer progression is intriguing.

Alternatively, LRG1 may be playing a role in apoptosis. We have found that MCF-7 breast cancer cells transfected with *LRG1 *are more resistant to apoptosis induction than non-transfected cells due to cytoplasmic LRG1 binding cytochrome *c *and inhibition of Apaf-1 activation (Jemmerson and colleagues, manuscript in preparation). In addition, transformed granulocytic cells transfected with *LRG1 *were reported by Ai et al. [21] to be more viable than non-transfected cells when transferred between different media. Likewise, LRG1 may be a survival factor for ovarian cancer cells, possibly rendering them more resistant to chemotherapy. It is interesting to note that the cisplatin-resistant A2780-CP cells express higher levels of LRG1 protein than their more sensitive counterparts A2780-S (Figure 3B); however, no difference in LRG1 protein expression was found for the cisplatin resistant cell line C13, compared to the corresponding cisplatin sensitive cell line OV2008.

## Conclusions

We have demonstrated the potential for using LRG1 as a serum biomarker for ovarian cancer. Furthermore, we showed the expression of *LRG1 *mRNA and protein in ovarian cancer tissues and cell lines, signifying that the tumor cells could be contributing to the increased levels of LRG1 in sera of ovarian cancer patients. Though future studies using a larger patient cohort are needed to determine whether LRG1 may serve as a biomarker for presurgical diagnosis of ovarian cancer, for the detection of recurrent disease, and/or as a target for therapeutic treatment, these initial results are encouraging.

## Competing interests

R.J. holds U.S. Patent 7,416,850 B2 for the LRG1 ELISA employed in this study. Although he supervised the assaying, he did not handle the samples and did not know their identification until the data were tabulated.

## Authors' contributions

JA performed the Western blots, immunocytochemistry, and conditioned media experiments, participated in the design of the study and data analysis, and drafted the manuscript. RJ designed and supervised the ELISA assay, and performed the glycosidase assay. KB participated in the data analysis and writing of the manuscript. PA and MG participated in the design of the study, oversaw the collection of patient samples, and edited the manuscript. BW participated in the design and analysis of the identification of LRG1 in conditioned media, and performed the mass spectrometry. BM and SW performed the ELISA experiments. KH performed the RT-PCR analysis. RI performed the data analysis and helped to draft the manuscript. AS participated in designing, coordination and supervision of the study, and writing of the manuscript. All authors read and approved the final manuscript.
